# Occupational issues of adults with ADHD

**DOI:** 10.1186/1471-244X-13-59

**Published:** 2013-02-17

**Authors:** Marios Adamou, Muhammad Arif, Philip Asherson, Tar-Ching Aw, Blanca Bolea, David Coghill, Gísli Guðjónsson, Anne Halmøy, Paul Hodgkins, Ulrich Müller, Mark Pitts, Anna Trakoli, Nerys Williams, Susan Young

**Affiliations:** 1 South West Yorkshire NHS Partnership Foundation Trust Manygates Clinic, Portobello Road, WF1 5PN, Wakefield, UK; 2 Leicestershire Partnership NHS Trust, London, UK; 3 King’s College London, Institute of Psychiatry, London, UK; 4 Department of Community Medicine, College of Medicine & Health Sciences, UAE University, Al Ain, United Arab Emirates; 5 Trincay Medical Centre & Urgent Care, George Town, Cayman Islands; 6 Division of Neuroscience, Medical Research Institute, University of Dundee, Ninewells’ Hospital and Medical School, London, UK; 7 Department of Biomedicine, Psychiatry, University of Bergen (UiB), Bergen, Norway; 8 Shire Development LLC, PA, Wayne, USA; 9 ADHD Service, Cambridgeshire & Peterborough NHS Foundation Trust, Cambridge, UK; 10 South London and Maudsley NHS Foundation Trust, London, UK; 11 Bradford Teaching Hospitals NHS Foundation Trust, Bradford, UK; 12 Independent Consultant in Occupational Medicine, Solihull, UK

## Abstract

**Background:**

ADHD is a common neurodevelopmental disorder that persists into adulthood. Its symptoms cause impairments in a number of social domains, one of which is employment. We wish to produce a consensus statement on how ADHD affects employment.

**Methods:**

This consensus development conference statement was developed as a result of a joint international meeting held in July 2010. The consensus committee was international in scope (United Kingdom, mainland Europe, United Arab Emirates) and consisted of individuals from a broad range of backgrounds (Psychiatry, Occupational Medicine, Health Economists, Disability Advisors). The objectives of the conference were to discuss some of the occupational impairments adults with ADHD may face and how to address these problems from an inclusive perspective. Furthermore the conference looked at influencing policy and decision making at a political level to address impaired occupational functioning in adults with ADHD and fears around employing people with disabilities in general.

**Results:**

The consensus was that there were clear weaknesses in the current arrangements in the UK and internationally to address occupational difficulties. More so, Occupational Health was not wholly integrated and used as a means of making positive changes to the workplace, but rather as a superfluous last resort that employers tried to avoid. Furthermore the lack of cross professional collaboration on occupational functioning in adults with ADHD was a significant problem.

**Conclusions:**

Future research needs to concentrate on further investigating occupational functioning in adults with ADHD and pilot exploratory initiatives and tools, leading to a better and more informed understanding of possible barriers to employment and potential schemes to put in place to address these problems.

## Background

Attention Deficit Hyperactivity Disorder (ADHD) is characterised by pervasive symptoms of hyperactivity, inattentiveness and impulsivity [1]. These symptoms surface relatively early in childhood and are fairly persistent over one’s lifespan [2]. ADHD is a condition that translates itself as overt behavioural disturbances in pre-school infants, school age children, adolescents and adults. Prevalence estimates are highly dependent on three main factors: the population sampled, the method of ascertainment, and the diagnostic criteria applied. The reported prevalence of ADHD in school age children varies from 1.7% to 17.8% depending on the criteria used [3]. Most estimates lie between 5% and 10% [4]. US estimates have historically been higher than UK estimates, due presumably to the application of narrower diagnostic criteria by UK authors [5]. Three studies of English populations have shown a prevalence rate of between 2% and 5%, depending on whether DSM-IV or ICD-10 criteria were applied [4,6]. ADHD has been found to not only be prevalent in the West but also in the Middle East, Africa and Asia [7]. Co-morbid Anxiety Disorders, Mood Disorders and Substance Misuse are commonly reported [8,9].

Research on the aetiology of ADHD has concentrated on three distinct strands: morphological differences, hereditary factors and functional differences. *Morphological differences* has been reported in children with ADHD for some time and volumetric [10,11] and cortical thickness [12] studies, identified abnormalities in the dorsolateral prefrontal cortex (DLPFC), the frontoorbital cortex, the anterior cingulate cortex (ACC), the inferior parietal lobule (IPL), and the corticostriatal system. The estimated *heritability* of ADHD from simple genetic analyses ranges from 0.6 to 0.9 [13] and an association between dopamine system genes and attention deficit hyperactivity disorder has also being shown [14].

*Functional abnormality* in people with ADHD has also been documented in multiple domains; it is therefore suggested that ADHD is neuropsychologically heterogeneous [15,16]. However, these deficits are closely linked with the ability to function at work [17-19]. Both children and adults experience motor co-ordination problems, which in adults could be detrimental to employment. Adults with ADHD also experience problems with working memory, planning and anticipation [20,21]; they may also experience issues in verbal fluency, effort allocation, application of organisational strategies and self-regulation of emotional arousal.

Despite this evidence, the general public associates ADHD with clear representations of a rampant child, rowdy and disobedient; brought on by the consumption of chemical additives. There is no doubt that ADHD has been under the spotlight of public scrutiny for a while, the majority of which has been negative. This publicity has resulted in defective and flawed representations of ADHD, and a disregard of Adult ADHD by some practitioners. It is our opinion that this neglect has resulted in policy makers, health professionals and businesses in general overlooking the occupational difficulties that can arise with ADHD. If we are to put in place the relevant framework and treatments to address these problems, Psychiatry, Occupational Medicine and Policy Makers must build bridges and focus on the needs of adults with ADHD and the barriers in employment that they face.

### Effects of adhd at the workplace

The 'workplace' is a complex personal and social construct and indeed, occupation has been defined as “active process of living" [22]. Models of human occupation have been studied using systems theory which refers to and was originally based on concepts of open systems and general systems theory [23,24]. More recently, dynamical systems theory [25], lead to the construction of the principle of “heterarchy” which suggests that aspects person’s environment are linked into a dynamic whole [26,27] and that each component contributes something to a total dynamic. These aspects of a person's environment are affected by adult ADHD.

In general, most employees in the second half of the 20th century, are paid for their “clock time” rather than specific outcomes with most of jobs designed as “mechanical” cogs in a larger production apparatus. It is in these “organisation ran by rules,” [28] that adults with ADHD are requested to operate and this 'fit' is not always successful.

ADHD is seldom diagnosed as a single disorder. In fact for patients with no diagnosis of ADHD, it is the co morbid that will lead the person present for help [29]. Co morbid disorders are common in adults with ADHD and include substance use disorders [30], depression [31], anxiety disorders [32], personality disorders [33] and of these specifically antisocial personality disorder [34-36]. In terms of the mood symptoms, these may be may be better understood as a core feature of the ADHD syndrome [37]. Furthermore, adults with ADHD may also present with ‘hidden impairments’ a term which includes ADHD, Autism Spectrum Disorders, Dyspraxia, Dyslexia and Dyscalculia [38] so consideration should be given in diagnosing those disorders as well.

The majority of research on adult ADHD has been conducted in the USA, and few studies have examined implications for social and occupational functioning. The fact that ADHD is associated with work-related problems in adulthood such as poor job performance, lower occupational status [39], less job stability [40] and increased absence days in comparison to adults without ADHD [41] has been documented. With the current vogue for health economic arguments, one would have expected a stronger representation in the literature. It seems that this argument is just beginning to be studied and in the future further studies will emerge.

In comparison to clinical experience about adult ADHD, empirical evidence about the expression of the disorder and the impairment it creates is relatively sparse. A few longitudinal studies following children with ADHD into adulthood have documented impairment in scholastic, occupational, relationship, and daily life functioning [33,42-45] and this impairment is not different between genders [46].

As expected, outcomes from studies of adults diagnosed with ADHD in childhood and followed to adulthood, differ somewhat from findings with samples of clinic-referred adults [47] with clinic-referred adults reporting greater functional impairment than controls. Although studies of clinic-referred adults with ADHD indicate increased psychiatric comorbidity [36], clinic-referred adults may report even higher rates of internalising symptoms than adults followed from childhood [17]. Additionally, clinic-referred adults with ADHD and adults reporting a prior ADHD diagnosis have more relationship and employment problems than controls [48,49] and describe themselves as less socially competent [50].

The poor performance and work loss for adults with ADHD is likely to have profound economic implications. One study quantified this impact by estimating the excess costs (i.e. the difference between adult ADHD patients and matched controls) related to work loss [44]. Indirect work loss costs were calculated based on employer payments for disability claims and imputed wages for medically-related work absence days (e.g. days in the hospital, physician visits). Another study estimated that adult ADHD was associated with a 4-5% reduction in work performance (chi12=9.1, p=0.001), a 2.1 relative-odds of sickness absence (chi12=6.2, p=0.013), and a 2.0 relative-odds of workplace accidents-injuries (chi12=5.1, p=0.024) [51]. The excess costs were $1.20 billion for women with ADHD and $2.26 billion for men with ADHD in this US Study [52]. However, after controlling for substance abuse, history of depression or anxiety, it was stimulant therapy during childhood that was the strongest predictor for being in work as adults (odds ratio = 3.2, p = .014). In this study, 414 patients responded to questionnaires rating past and present symptoms of ADHD, co morbid conditions, treatment history, and work status [44] it was suggested that treatment of ADHD in childhood created a pathway to employment.

A recent survey undertaken by the World Health Organisation in 10 countries reported that 3.5% of the workers suffered from ADHD resulting in 143 million days of lost production. Workers with ADHD had an average 8.4 excess sickness absence days per year and even higher annualised average excess numbers of work days associated with reduced work quantity (21.7 days) and quality (13.6 days) Furthermore, a small minority of these workers are treated for ADHD despite evidence that such treatment can be quite effective in improving functioning [53].

### Effects of adhd at separate stages of employment

#### The job application

The Consensus identified that ADHD adults experience impairment in all aspects related to employment, from the initial job search, to the interview and then in employment itself. When searching for a job they are disorganised and sporadic and find it difficult to interact with Employment Advisors at Job Centre. This difficulty in creating a working alliance with advisors most likely stems from the fact that adults with ADHD are disenfranchised and disengaged from the system in general. For example, their attentional problems and poor understanding of social conventions results in them avoiding ‘tedious’ tasks such as writing detailed application forms, completing them by missing out items and/or completing them without having read the question properly or reflected on their answer.

#### The job interview

At the interview the person may come across as very friendly and chatty. They may be a little inaccurate about their past history, and may overstate their ability to fulfil the requirements of the job. This is not the sole mandate of people with ADHD, nevertheless it is characteristic of many hopeful interviewees! An important problem however, is the decision of whether to disclose their ADHD status on application forms or in interviews. Most employers do not understand the implications of ADHD. They panic that it is a profound and prolonged condition and are not aware that it can be effectively treated and that reasonable adjustments can be easily made that will improve occupational functioning.

#### The working environment

Once employed and initially in post, ADHD adults may be highly motivated workers but, depending on the job, ADHD symptoms soon begin to hamper the person’s performance. Some people find functional employments that mask organisational problems (e.g. where they have secretarial or administrative support), or have employment that complements their symptoms (e.g. highly creative work or sports). Nevertheless, most skilled and unskilled occupations (e.g. administration posts) will be hampered by symptoms of inattention, impulsivity and hyperactivity.

Employees may have difficulties with time management, organising their schedule, keeping on top of their work load, following instructions and exhibit emotional liability. These are important symptoms to look out for when assessing the level of impairment above and beyond the norm. Individuals with ADHD may also be disadvantaged by poor social skills and procrastination, which make it hard for them to work effectively with colleagues, accept line management, and/or deal with the public. In trying to resolve the issue around lack of performance a line manager will most likely confront the employee, this critique will most likely be taken personally and from then on it will be difficult to re-motivate the person. Relationships with colleagues will become impulsive, abrasive and volatile. Information relayed in job appraisals will not be ensued and acted on as they are not in a position to adapt their behaviour. This most likely will result in another lost employment.

Adults with ADHD can also become hyper-focused in activities especially if they are incentivised by it. Thus it is important to be aware of the potential for workaholism in adults with ADHD. An employer must harness the positives of ADHD and maintain the right balance, taking care to avoid burnout.

Further research is required in order to fully understand the factors that influence occupational impairment and to develop specific and effective interventions. Identifying and treating ADHD in childhood is likely to improve long-term clinical and occupational outcome. Addressing co-morbidity is essential as this will further impair a patient’s functioning. It is vital that clinicians, when assessing occupational functioning of an adult, have a concise and detailed understanding of the situation they are in and to follow treatment outcome. This will facilitate the best treatment option, e.g. psychological or occupational therapy.

### Occupational health perspective

#### Screening

The discussion above raises the question whether adult ADHD is a candidate for targeted work place screening and treatment programs. The argument is that ADHD among workers has non-trivial prevalence, high impairment and a low rate of treatment, whereas cost-effective therapies exist that are related to improvements in some objective aspects of role performance [54,55]. In the first instance therefore, as part of the pre-employment screening, scales such as the adult ADHD self-report scale (ASRS) [56] can be used routinely to identify potential employees with ADHD. are management and Human Resources must refer people with ADHD to Occupational Health Physicians need to be equipped and well-informed to be able to recognise these needs and suggest reasonable adjustments.

#### Adjustments

There are several adjustments that could accommodate the needs of adults with ADHD. These need not be expensive or time-consuming to organise. Generally managers should implement systems that include regular meetings, reviews, and structured feedback for the individual. Verbal information should be supplemented in written format with clear, concise instructions. More specifically, to optimise attention and reduce distraction, employees could be provided with a private office, located in quieter rooms or positioned to reduce likelihood of distraction. They could also be offered flexi-time to work at quieter times of the day, or provided with headphones to reduce external noise. Impulsivity could be accommodated by a buddy system, as teaming up with a colleague will help the individual to remain focused. Hyperactivity can be dealt with by allowing productive movements at work, encouraging staff to remain active, and ensuring structured breaks are included in long meetings. Adults with ADHD suffer with organisation, time management and memory problems, and employers can tackle these problems by providing alarms or beepers, the use of structured notes and agendas, and providing regular supervision or mentoring, delegating more tedious tasks such as paperwork, and/or introducing incentives and rewards for task-completion. Best practice is one that identifies the problem and then successfully implements reasonable adjustments (Table 1).

**Table 1:** Potential workplace adjustments for adults with ADHD

**Symptom**	**Possible adjustments**
Attention and impulsivity	Private office/quieter room/positioning in office, flexi-time arrangement, headphones, regular supervision, buddy system.
Hyperactivity/restlessness	Allowing productive movements at work, encouraging activity, structured breaks in long meetings.
Disorganisation, time management, and memory problems	Provide beepers/alarms, structured notes, agendas, regular supervision with frequent feedback, mentoring, delegating tedious tasks, incentive/reward systems, regularly introducing change, breaking down targets and goals, supplement verbal information with written material.

#### Vocational Rehabilitation

Taking the view that vocational rehabilitation is whatever helps someone with a health problem to stay at, return to and remain in work [57] the standard basic principles of healthcare and workplace management should apply for adults with ADHD. These are based on two functions: first, on *healthcare which includes a focus on work*, incorporating the idea of vocational rehabilitation, early intervention, and intervention tailored to individual needs and second, *workplaces that are accommodating* incorporating a proactive approach to supporting return–to-work, and the temporary provision of modified work and accommodations. Currently, this approach is not applied for adults with ADHD in the same manner as it is applied for people with common health problems such as anxiety, depression, stress, musculoskeletal pain and this places them at immediate disadvantage.

### Health policy perspective

The United Kingdom Government approach to employment in relation to mental ill health did not start from a medical perspective but from a sociological perspective. In 2004, the Social Exclusion Unit published a report [58] which brought together analysis to show that people with mental health conditions were one of the most excluded groups in society. Having the opportunity to employment was seen as one of the routes to social inclusion and as a result, the Government commissioned more work to inform employment policy. Approximately 5 years later, almost concurrently, a number of reports were published which first, developed the framework for wellbeing at work for all and better employment results for people with mental health conditions in and out of work [59], second, set out a series of actions [60] on how there can better help more people with mental health conditions who are workless into sustained employment [61] and third, recognised the importance of work to the journey of recovery from mental health condition [62]. Although these reports are laudable in their scope, they still missed the opportunity to include a number of disadvantaged individuals who do not always fall within the conversional mental illness definitions such as adult ADHD.

A general review of the health of Britain’s working age population produced a document called ‘*Working for a Healthier Tomorrow*’ [63] (Table 2) which highlighted the need to swiftly change the widespread view that someone has to be 100% fit in order to be in work and made a number of recommendations which also apply for adults with ADHD.

**Table 2:** Key recommendations & findings from the Dame Carol Black report (2008)

**Number**	**Key recommendations & findings**
1	Supporting the health of working age people requires the co-ordination and integration of a range of professional disciplines.
2	Strong support needed for health and wellbeing initiatives in the workplace reinforced by visible management commitment.
3	The importance of physical and mental health of working age people is insufficiently recognised in society, there is a need for the Government to launch a national awareness raising campaign.
4	A need to change the view that people need to be 100% fit in order to be in work and the introduction of a ‘fit note’ which should concentrate on what people can do rather than what they can’t.
5	The importance of early intervention to reduce number of people on long term sick leave or incapacity benefit.

Currently employers are ill-informed and wary of recruiting disabled people, resulting in negative attitudes and defensive recruitment practices. Therefore it is important to encourage employers to think positively about the benefits of recruiting disabled people, who are reported to stay in employment longer and do a better job. More importantly employers are oblivious to disabilities such as ADHD and therefore fail to pick up on crucial symptoms or even differentiate between ADHD and non-ADHD.

### What next?

Partnership working will be essential to address occupational functioning in adults with ADHD. Input from occupational health professionals, employers, government, clinicians, voluntary sector and service-users will help create an all inclusive solution focused directive.

In order to maximise the employment and retention of adults with ADHD, it is imperative that, with the support of the Government, local strategic partnerships and employers develop tools for employers to better understand ADHD as a disability. Raising awareness amongst small and large businesses will result in employers feeling more confident about the skills, limitations, and ‘employability’ of adults with ADHD. The provision of vocational rehabilitation for adults with ADHD may reduce occupational dysfunction and co-morbid conditions, such as depression, anxiety. In particular, specific interventions could be developed to address core problems such as inattention, impulsivity, hyperactivity, distractibility, disorganisation, and time management problems in occupational settings.

As the recommendations of the Black review [63] become reality, there will be a need to educate policy makers and clinicians such as GP’s to the functional difficulties faced by adults with ADHD and how specific initiatives can be developed. There is an opportunity here for ADHD interest groups to develop guidance for GP’s in completing ‘Fit Notes’ for adults with ADHD.

There is a lack of research on the topic of employment of adults with ADHD and in general, a lack of understanding on how to address these occupational issues still remains. Future research should focus in integrating findings using the Vocational Rehabilitation principles. As a first step, specialist occupational therapy interventions need to be developed [64] specifically addressing the needs of adults with ADHD.

## Competing interests

Dr P Hodgkins is an employee of Shire Development LLC. He also owns stock/stock options in the company. Shire develops are markets drugs to treat psychiatric disorders including ADHD. MA, SY, DC, MA, PA, GG and UM have received pharmaceutical funding for consultation, speaking, and/or conference support.

## Authors’ contributions

DC, MP and SY have been involved in drafting the manuscript and revising it critically. All authors have given final approval of the version to be published.

## Pre-publication history

The pre-publication history for this paper can be accessed here:

http://www.biomedcentral.com/1471-244X/13/59/prepub
